# Cost-effectiveness analysis of two routine therapeutic methods for *Helicobacter pylori* eradication: a Persian cohort-based study 

**Published:** 2021

**Authors:** Farhad Pourfarzi, Telma Zahirian Moghadam, Hamed Zandian, Reza Malekzadeh, Abbas Yazdanbod

**Affiliations:** 1 *Digestive Diseases Research Center, Ardabil University of Medical Sciences, Ardabil, Iran*; 2 *Social Determinants of Health Research Center, Ardabil University of Medical Sciences, Ardabil, Iran*; 3 *Social Determinants of Health Research Center, Ardabil University of Medical Sciences, Ardabil, Iran*; 4 *Digestive Oncology Research Center, Digestive Diseases Research Institute, Shariati Hospital, Tehran University of Medical Sciences School of Commerce, Tehran, Iran *; 5 *Digestive Diseases Research Center, Ardabil University of Medical Sciences, Ardabil, Iran *

**Keywords:** Cost-effectiveness, Helicobacter pylori, Eradication, Furazolidone, Clarithromycin

## Abstract

**Aim:**

This study aimed to analyze the cost-effectiveness of two routine therapeutic methods for *H. pylori* eradication in Iran

**Background:**

Because of the importance of *Helicobacter pylori (H. pylori)* eradication on gastric cancer prevalence and costs, an economic analysis of the eradication methods is essential for health systems.

**Methods:**

This cross-sectional study was conducted on 7,496 participants with positive Hepadnaviridae (HPsAg) test results for *H. pylori*; 6,163 of them were treated with furazolidone (group A), and 1,333 participants were treated with clarithromycin (group B). Data on GP visits, medications, and HPsAg costs as direct costs and absence from work and transportation as indirect costs was collected by researcher-made questionnaire. Indirect costs were calculated based on face-to-face interviews with 365 patients of the Persian Cohort Center. Successful eradication of *H. pylori* infection (negative HPsAg) was defined as the effectiveness of the interventions. Incremental cost-effectiveness ratio (ICER) was used to compare the overall results.

**Results:**

The total direct cost of *H. pylori *for groups A and B were estimated at 13.7 and 5.83 billion IRR, respectively. The highest and lowest percentages of total costs were the cost of diagnostic services and the time cost, respectively. There was a significant difference between the two groups in drug costs (*p*<0.001). The effect ratio for groups A and B was 85.93% and 96.54%, respectively. Cost per effectiveness was higher for clarithromycin (CE=3,250,170 IRR) than for furazolidone (CE=2,988,488 IRR), and ICER showed that 5.1 Million IRR per participant is needed to eradicate *H. pylori*.

**Conclusion:**

Based on the results, furazolidone was more cost-effective than clarithromycin for *H. pylori* treatment. Therefore, due to the high prevalence of *H. pylori* and the economic conditions of the health system in Iran, furazolidone can be a cost-effective choice between the two conventional treatment methods considering the results of further research and possible side effects.

## Introduction

*Helicobacter pylori (H. pylori)* is a gram-negative micro-aerophilic bacterium that infects the epithelial lining of the stomach and is known as the leading risk factor for gastric cancer (1, 2). Several studies have shown that *H. pylori* is the main cause and principal etiological agent for gastric cancer and peptic ulcer disease (3). In various regions of Iran, the frequency of adult-onset over 2 years has been reported to be around 2%. Spontaneous bacterial remission may be relatively common in childhood, but *H. pylori* infection in adults is usually persistent and will not improve without specific treatment (4).

Although the incidence of *H. pylori* infection has been decreasing in association with improved standards of living, the prevalence of this bacterium is still ubiquitous, especially in developing countries (5). This infection affects more than 50% of the population, and its frequency varies considerably between population groups in each particular country (6).In developing and developed countries, its prevalence is 90% and 30-50%, respectively (5, 7). The prevalence remains high in most developing countries and is generally related to socioeconomic status and levels of hygiene (8). *H. pylori* has infected more than half of the Iranian people during the last decade, where it is estimated to affect all Iranian citizens, children, and adults at rates of 54%, 42%, and 62%, respectively (9).

The globally increasing growth of healthcare costs has become one of the main concerns of health system managers and decision-makers. The continued expansion of new and expensive health technologies, increased societal expectations of health systems, and the rise of chronic and severe diseases among the population are important reasons for such rapid growth (10). Similar to all other health systems, the Iranian health system also faces the challenge of dramatically increased costs (11, 12). While the overall expenses index has become 30 times greater in Iran within the last twenty years, growth in healthcare expenses has seen an increase of 71 times. As a developing country that is plagued by many economic problems, saving healthcare costs can reduce these problems to some extent. Hence, the government is looking for a way to reduce the economic burden on the healthcare sector (13). 

Gastric cancer is the most conventional and highly prevalent gastrointestinal cancer in northwestern regions of Iran. Unlike western countries and Japan, the incidence of gastric cancer in Iran has increased over the past two decades (14). The highest average of gastric cancer incidence in the world (ASR of 51.8/100,000 in men and 24.9/100,000 in women) is found in Ardabil, a volcanic and mountainous region in the northwestern province of Iran, located in the Caspian Sea littoral, where *H. pylori* positivity has been seen as a significant determinant of the risk of gastric cancer (15, 16). The rate of gastric cancer in Ardabil is still rising despite the worldwide pattern recorded, with age-standardized incidence rates of 49.1 and 25.4 per 100,000 for males and females, respectively (17). Several risk factors may play a role in the high incidence of gastric cancer in Ardabil, which has the highest GC mortality rate in Iran. Several tests have shown that in Ardabil, the *H pylori* prevalence rate is approximately 90% (18). This high rate has led to an increase in healthcare costs due to the increased number of patients affected and increase in unit costs (19). Moreover, it shows the importance of evaluating the cost-effectiveness of eradicating *H pylori* in this province to reduce the costs of gastric cancer. Primary prevention through *H. pylori* eradication is necessary, especially in countries such as Iran and areas like Ardabil, where the incidence of gastric cancer is high and large numbers of people are infected by *H. pylori* (20).

Several studies have shown that the eradication of *H. pylori* would prevent the recurrence of most related diseases and decrease the mortality rate of gastric cancer (21-23). The Guidelines of Maastricht V/Florence suggests culturing *H. Pylori*, antimicrobial susceptibility monitoring, and antibiotic selection depending on the effects of resistance studies (24). Based on the Maastricht II treatment scheme, Altintas et al. showed that there was no difference in *H pylori* eradication with omeprazole, lansoprazole, or pantoprazole, and the eradication rate was as low as 45% (25). 

Some other studies have shown that screening was likely to be cost-effective in high-income countries (26), but there is little evidence about low- and middle-income countries such as Iran. In their retrospective study, Kajihara et al. reported that as a first-line treatment for *H. Pylori* eradication, triple therapy based on vonoprazan was more cost-effective than rabeprazole (27). Hajaghamohammadi et al. showed that instead of clarithromycin, low-dose furazolidone could be used as a low-cost and efficient medication to eliminate *H. pylori*, with amoxicillin and omeprazole in combination (28). In contrast, Dong-Min et al. showed that despite the growing opioid resistance to clarithromycin,* H. Pylori* eradication rates in furazolidone and clarithromycin groups currently have no substantial difference in terms of cost-effectiveness (29). In Iran, it seems that the best first-line treatment for the eradication of quadruple therapy is based on clarithromycin or furazolidone administered for at least two weeks (30). Comparisons of the results of the two treatments in different studies, however, have revealed different results, both in terms of effectiveness and cost. Accordingly, the current study was conducted to compare the cost-effectiveness of two therapeutic methods (clarithromycin and furazolidone) in the eradication of *H. pylori* based on data from a Persian cohort study as first-line treatment in adults. Importantly, the present study included indirect medical and non-medical costs to evaluate the cost-effectiveness of the two routine treatment methods, which have not been considered in most studies. 

## Methods

**Study design and setting**

The present work is a cross-sectional economic evaluation study for estimating cost-effectiveness and comparing the two medicinal groups of clarithromycin and furazolidone. The study sample was selected from residents over 35 years of age in Ardabil city (the capital city of Ardabil province in northwestern Iran), who were present in a Persian cohort study from 2017 to 2019.

**Eligibility Criteria**

Chief candidates for this study were patients diagnosed with *H. pylori* infection over the age of 35 years. Individuals with a positive *H. pylori* test result, who were willing to participate in the study, non-pregnant women, those not diagnosed with non-cardia gastric cancer, intestinal- or diffuse-type gastric cancer, or a history of *H. pylori* treatment, and those not taking medication related to psychiatric disorders (for the prevention of drug interactions) were randomly enrolled.

**Sampling method and sample size**

The samples were selected from the Persian cohort registrants living in Ardabil (with a population of 18,000). First, a list was prepared of all Persian cohort registrants and their logged clinical records. Then those meeting the inclusion criteria, primarily those with a positive stool exam result for *H. pylori*, were identified to participate in this study (a population of 7,496). The two mentioned groups were classified as groups A and B as follows: A) 6,163 participants treated with furazolidone (i.e. amoxicillin 500 + omeprazole 20 + bismuth 120 + furazolidone 100), and B) 1,333 participants treated with clarithromycin (i.e. amoxicillin 500 + omeprazole 20 + bismuth 120 + clarithromycin 500) and treated for two weeks. 

A pilot study was conducted in face-to-face interviews with 365 patients of the Persian Cohort Center, which was calculated by Cochran's sampling formula, for a specific population of participants with *H. pylori *to calculate the indirect costs. Single-month indirect cost-related data was extracted from a checklist throughout participation in the Persian cohort and *H. pylori *intervention.

**Data Collection**

A researcher-made questionnaire completed in interviews with participants was used to collect data from May 2017 to February 2020 on participant age, gender, time spent in the Persian Cohort Center, and additional relevant costs such as physician's visits, diagnostic services, transportation, absence from work, and medications. The questionnaire consisted of two sections: the first comprised demographic information (i.e. age, gender, marital status, education status, number of family members, income, and history of illness). The second section included information on direct costs (general practitioner's visits, average prices of medications used (primary and complementary medications), and necessary tests (e.g., HPsAg)) as well as indirect costs (absence from work and transportation).

To determine effectiveness, the efficacy analysis of therapeutic interventions was undertaken based on successful eradication of Helicobacter infection as a negative Hepadnaviridae (HPsAg) result. For this purpose, participants in both groups A and B received their drug combination for two weeks. In group A, the drug combination consisted of twice daily (BID) doses of amoxicillin 500, bismuth 120, furazolidone 100, and one dose per day (OPD) of omeprazole 20. The drug combination for group B differed only in OPD of clarithromycin 500 instead of furazolidone 100 every 12 hours. Two months after the end of drug therapy (two weeks), participants were re-tested in the Persian Cohort Center of Ardabil. The relative percentages of negative HPsAg results were reported as the effectiveness of the intervention in all treated individuals in each group.

$\mathrm{Effectiveness}=\frac{\mathrm{Number of negative test results}}{\mathrm{total number of drug users}}\times 100$

**Study variables and data sources**

Several data sources were used to estimate the cost-effectiveness of *H. pylori *drug therapy in the present study. The most important source used to estimate direct costs was the data extracted from patient cases of positive H. pylori test results in the Persian Cohort Center of Ardabil. 

This study analyzed costs from two perspectives: society (including sick-leave cost of output loss) and the healthcare sector alone. Methods in health economic assessment compare the incremental expense against a treatment's incremental health gain (31). The study perspective to direct and indirect costs was based on means of costs during treatment. All costs were calculated based on the Iranian currency (rials) and adjusted for comparability in terms of exchange rate each year during 2017 to 2020. Missing data, less than 5%, was corrected according to the mean cost and, if more than 5%, was removed.

***Direct Medical Costs***

Direct medical costs were attributable to the expenses of receiving *H. pylori *treatment services in the Persian Cohort Center, including general practitioner's visits, HPsAg, prices of the medications furazolidone (group A) or clarithromycin (group B) along with amoxicillin 500 + omeprazole 20 + bismuth 120 .

***Direct Non-medical Costs***

This group comprised costs incurred by the participant and his family, such as lost time. This period was extracted from the participant's records in the Persian Cohort System. The time cost was calculated and extracted by multiplying days off from work based on the daily average wage. Accordingly, employed people were asked about their average monthly income. For those who did not declare a specific monthly income or for housewives, this figure was calculated by the minimum wage amount declared by the Iranian Ministry of Labor, Cooperatives and Social Welfare, in 2018. Finally, the time cost for participants was calculated by multiplying the average daily wage by the average time spent at the center receiving relevant services. Other information, such as direct costs, was calculated based on the latest prices of drugs, tests, and visitation services declared by the Iranian Ministry of Health and Medical Education.

***Direct Non-medical Costs***

Transportation to receive services at the Persian Cohort for treatment of *H. pylori *infection was considered as an indirect cost in this study. Other direct non-medical costs such as care provided by friends and family, housekeeping, social services, etc. was not included in analysis due to the lack of this data for the majority of patients.

**Data analysis**

Following the determination of costs (direct and indirect) and estimation of effectiveness (negative HPsAg result for H. pylori), statistical methods were used for data analysis. The average cost was specified per medical and diagnostic service based on the prices declared by the Iranian Ministry of Health and Medical Education. Accordingly, the total cost was calculated by multiplying the average cost by the number of participants in each group. Percentage of total costs was calculated by dividing the total cost of each service unit by the sum of total costs. Independent t-test was used to compare the costs between the two groups. Effectiveness was estimated from the ratio of treated participants to the number of those receiving drugs and intervention in each group. For this purpose, the percentage of subjects with negative HPsAg result for *H. pylori *was calculated for all drug recipients in both groups. Then, the incremental cost-effectiveness ratio (ICER) was used to compare the overall results. The ICER was calculated according to the incremental cost required to treat a participant with *H. pylori *infection using the method of group A versus that of group B.

## Results

In total, 7496 subjects participated in the *H. pylori *infection treatment project, of which 47.4% were male and 52.6% were female. Among the members of groups A and B, the age groups of less than 40 years of age (18.6%) and the age group of 50-54 years (20.7%) had the highest frequency, respectively. The results showed no significant difference between the two groups in terms of gender as a demographic variable (*p*>0.05). Table 1 presents the demographic distribution of the participants in groups A and B separately. 

Data regarding direct medical costs (i.e. general practitioner visits, average prices of both primary and complementary medicines used) and necessary tests (stool and respiratory testing) was directly extracted from patients' case files, and data on indirect costs (transportation and absence from work) was extracted and estimated from interviews with patients. The mean total cost for participants in groups A and B was 2,570,100 IRR and 3,120,164 IRR, respectively. The total costs for groups A and B were 13,659,236,701 IRR and 5,839,578,803 IRR, respectively (as shown in Table 2). The mean difference in costs between the two groups shows that group B had higher mean expenses than group A by as much as 550,064 IRR. These costs were caused by *H. pylori *intervention.

**Figure 1:**
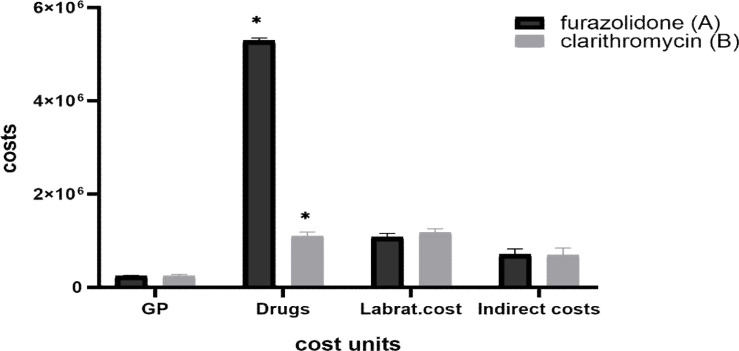
costs units for H-P elimination by two different methods A: patients treated with Amoxicillin 500 + Omeprazole 20 + Bismuth 120 + Furazolidone 100, and B: patients treated with Amoxicillin 500 + Omeprazole 20 + Bismuth 120 + Clarithromycin 500. There was no significant differences between two groups in terms of General Practitioner visit (GP), Laboratory costs (Labrat.cost), and Indirect Costs (included transportation and time costs). Nevertheless, there was a significant difference (*) between the two groups in drug costs (p<0.001), so that drug costs for group B was significantly higher than group A

The average cost difference in the four general categories of GP visits, medications and drugs, diagnostic services, and indirect costs is illustrated in Figure 1. According to the results, there was a significant difference between groups A and B in terms of medication and drug costs, and the other cost groups showed insignificant differences.

According to the number of individual subjects with negative HPsAg results in each group, the *H. pylori *eradication rates for participants in groups A and B were 85.93% and 96.54%, respectively. This result indicates the higher effectiveness of the group B intervention compared to that of group A. Cost-effectiveness analysis showed that for each subject undergoing *H. pylori *intervention in groups A and B, a cost of 2,988,488 IRR and 3,250,170 IRR, respectively, was paid (Table 3).

Given the results of Table 3, the ICER was estimated to be 5,184,392.08 IRR per *H. pylori *eradication. Accordingly, each ICER (eradication of H. pylori) by drugs of group B required 5,184,392.08 IRR more than that of group A. 

$\mathrm{ICER}=\frac{\mathrm{Mean Cost A}-\mathrm{Mean Cost B}}{\mathrm{Effec A}-\mathrm{Effec B}}=\frac{2570100-3120164}{85.93-96.54}=5184392.08$

**Table 1:** Baseline demographic characteristics of participants in two groups with H-P in north-west of Iran

Demographic characteristics	Group A (n=6163)	Group B (n=1333)	P-Value
Age mean (years)	49.11	50.87	<0.001
Age Groups, %			<0.001
<40	18.6	11.5	
40-44	18.1	14.2	
45-49	18.4	19.6	
50-54	16.1	20.7	
55-59	13.6	17.1	
+60	15.1	17	
Gender (%)			0.665
Female	52.1	53.2	
	47.9	46.8	

Group A: Amoxicillin 500 + Omeprazole 20 + Bismuth 120 + Furazolidone 100; Group B: Amoxicillin 500 + Omeprazole 20 + Bismuth 120 + Clarithromycin 500

**Table 2:** Average and Total Cost of two methods of H-Pylori elimination in Ardabil (North-west of Iran), 2018-2019

Service Item		Group A			Group B		
		Mean Cost	Total Cost	%	Mean Cost	Total Cost	%
Visiting by GP		245000	1,509,935,000	9.5	245000	326,585,000	7.9
Diagnostic tests	Pre intervention	545000	3,358,835,000	21.2	545000	726,485,000	17.5
	Post-intervention	545000	3,358,835,000	21.2	545000	726,485,000	17.5
Medication & Drugs	Amoxicillin Cap	173600	1,069,896,800	6.8	173600	231,408,800	5.6
	Omeprazole cap	61600	379,640,800	2.4	61600	82,112,800	2.01
	Bismuth tab	246400	1,518,563,200	9.6	246400	328,451,200	7.9
	Furazolidone tab	48000	295,824,000	1.9	-	-	-
	Clarithromycin tab	-	-	-	613200	817,395,600	19.7
Transportation Costs		198,650	1,224,279,950	7.7	178,650	238,140,450	5.7
Time costs (Absence from work)		506,850	3,123,716,550	19.7	511,714	682,114,762	16.4
Total Costs		2,570,100	13,659,236,701	100	3,120,164	5,839,578,803	100

Group A: Amoxicillin 500 + Omeprazole 20 + Bismuth 120 + Furazolidone 100; Group B: Amoxicillin 500 + Omeprazole 20 + Bismuth 120 + Clarithromycin 500

**Table 3:** cost effectiveness analysis of two different drugs to elimination of H-P in Persian Cohort participants in north-west of Iran, Ardabil 2018-2019

Cost-Effectiveness	Effect Ratio	Mean Costs	PoTS	NeTS	Total Subjects	
2,988,488	85.93	2,570,100	867	5296	6163	Group A
3,250,170	96.54	3,120,164	46	1287	1333	Group B

NeTS= Negative stool test for H-Pylori, PoTS= Positive stool test for H-Pylori, Effect Ratio= NeTS or PoTS / Total Subject, Cost-Effectiveness= Mean Costs / Effect Ratio

Group A: Amoxicillin 500 + Omeprazole 20 + Bismuth 120 + Furazolidone 100; Group B: Amoxicillin 500 + Omeprazole 20 + Bismuth 120 + Clarithromycin 500

## Discussion

The primary purpose of the present study was to investigate and compare the cost effectiveness of two treatment regimens of *H. pylori *eradication in Ardabil on participants in the Persian Cohort. This study is one of few that have examined the cost effectiveness of *H. pylori *treatments in terms of direct and indirect medical costs in Iran, which is urgently needed due to the high prevalence of *H. pylori *infection in Iran (32, 33). Eradication of *H. pylori *is considered a cost effective method in the face of gastric cancer incidence (21). Currently, numerous treatment regimens approved by the US Food and Drug Administration are being used to eradicate *H. pylori *(34). Among them, two treatment regimens, referred to as group A and group B in the present study, were routinely used in Ardabil health centers. Given the importance of treatment costs, especially in developing countries with a high prevalence of *H. pylori *infection such as Iran, the evaluation and identification of the most cost-effective ways to treat this infection in the population are paid much attention (35, 36). Also given that both A and B treatment methods are conventional approaches for *H. pylori *and a significant population needs to be treated, its cost effectiveness is essential from this perspective as well. In this study, effectiveness was considered and evaluated based on *H. pylori *treatment (complete removal of bacteria).

According to the results shown in Table 3, the effect ratio of group B (96.54%) was higher than that of group A (85.93%). Fatahi et al. (2016) showed the effectiveness of eradication of *H. pylori *in participants who received clarithromycin 500 mg twice daily, amoxicillin 1 g twice daily, and omeprazole 20 mg one to two weeks after the intervention to be 86% on average (37). Their findings regarding group B were lower than those of the present study. The difference in the duration of follow-up after intervention can be considered one of the factors affecting the differences in effectiveness in the two studies, as the participants in the present study were followed for two months, while Fatahi et al. followed their participants for only two weeks (2016). In another study conducted in China, participants were treated with omeprazole, amoxicillin, metronidazole, and bismuth for a week. The eradication rate in that study was 95%, which is in line with the findings of the present study (38).

In a study conducted in Finland, the effectiveness of a 2-week treatment with a four-dose regimen consisting of omeprazole, amoxicillin, metronidazole, and bismuth and a subsequent 2-week treatment with omeprazole was reported to be 100% (39). Treiber et al. (1996) reported the best method of eradicating *H. pylori *to be a 1-week treatment with omeprazole (20 mg daily), imidazole (e.g., metronidazole 400 mg BID), and clarithromycin 250 mg (BID) (36). In a randomized control trial, Riyahizadeh et al. showed that furazolidone could replace clarithromycin in *H. pylori* eradication regimens because of the lack of development of resistance and very low cost (40). However, they only considered the costs of treatment and no other costs in their study. Low-dose furazolidone was also recommended in another study from Iran as a low-cost and efficient medication for eliminating *H. pylori *(28).Furthermore, another study in Iran showed that treatment with three drugs, including a protein pump inhibitor, clarithromycin, and amoxicillin, is the most effective regimen in the first-line treatment of *H. pylori *infection. Triple therapy based on vonoprazan was more cost effective than rabeprazole in a study by Kajihara et al. (27). In cases of high drug administration, a quadruple therapy regimen can be used as an alternative, which is partially consistent with the effectiveness section of the current study (41). Overall, various studies have evaluated the effectiveness of different methods, including a combination of two antibiotics along with a PPI, to ensure complete eradication (26, 42-44) and have estimated the effectiveness of these methods to be approximately 90% on average. These findings are lower and higher than the results of the present study in groups B (the combination of clarithromycin with amoxicillin 500 + omeprazole 20 + bismuth 120 + furazolidone 100) and A (combination of furazolidone 100 with amoxicillin 500 + omeprazole 20 + bismuth 120), respectively, indicating the effectiveness of the intervention of group A in the present study was lower than most combination drug interventions.

In the present study, both direct and indirect costs were used to estimate the cost of *H. pylori *treatment. According to the results, the average cost of treatment of *H. pylori *with furazolidone (group A) was lower than that of clarithromycin (group B). However, there was no significant difference between the two groups in terms of the cost of visits, diagnostic services, time, and transportation (commuting); the main difference between the two groups was in the mean costs of drugs. Briggs et al. (1996) examined the cost effectiveness of *H. pylori *screening merely for direct costs and showed that the most significant cost differences in the two drug groups could be attributed to drug costs (45), which is in line with the findings of the present study. Gosh et al. (2012) calculated the costs associated with doctor visits, counseling, commuting, and laboratory during treatment and follow-up and then classified the total costs into two groups: direct and indirect costs. They showed insignificant differences between the three drug-taking groups in terms of indirect costs; significant differences were only caused by drug costs and the total cost of treatment (42), which is also in line with the results of the present study. Overall, different studies showed significant differences in the cost of treatment of *H. pylori *with the administration of drug groups only in terms of direct costs incurred for the drugs used to treat the disease itself and its corresponding side effects, which corroborates all findings of the present study (36, 46, 47). It should be noted that due to different drug interventions in diverse studies as well as differences in economic status and drug prices and *H. pylori *treatment remedies in various countries, there is no uniform basis for comparing costs in detail; costs are comparable only in terms of drug groups. 

The current findings showed that furazolidone 100 with amoxicillin 500 + omeprazole 20 + bismuth 120 (group A) was more cost effective compared to clarithromycin with amoxicillin 500 + omeprazole 20 + bismuth 120 + furazolidone 100 (group B). In other words, the subjects in group A paid a lower amount per treatment unit (effectiveness) compared with group B. The calculated ICER in the present study showed that for each one percent of greater effectiveness in group A compared with group B, a cost of approximately 5.2 million IRR is needed. Different studies have investigated the cost effectiveness of *H. pylori *treatment methods in terms of drug combinations. Omata et al. (2017) established a cost-effective diagnostic method for patients with atrophic gastritis and demonstrated that the ICERs of serum *H. pylori *IgG antibody (SHPAb), rapid urease test (RUT), and urea breath test (UBT) vary. However, due to the low effectiveness of the rapid urease test method, complete replacement of this method should be undertaken with caution compared to other methods, despite their lower costs (22). This result can be in line with findings of the present study in terms of the low cost effectiveness of group A, because here, group A had lower cost effectiveness compared with group B, but its effectiveness was significantly lower than group B, which casts doubt on using this method. Han et al. (2019) investigated the cost effectiveness of *H. pylori *treatment using the Marco model in both treated and untreated groups of patients with gastric cancer and showed that *H. pylori *treatment would bring a cost savings of $1539 per person and $168.45 per quality-adjusted life year (QALY) (21). Seko et al. (2019) evaluated the cost effectiveness of lansoprazole and vonoprazan and showed that the effectiveness of the two drugs was 75.2% and 87.8%, respectively. The cost-effectiveness of the lansoprazole group was higher than that of the vonoprazan group. However, they made no recommendation on the treatment method choice because of the significant difference in the effectiveness of the two drugs (23). Ten studies from different countries have used a four-drug regimen called OBNA, i.e. omeprazole, bismuth, nitroimidazole, and amoxicillin, with different durations of treatment. In this meta-analysis, the most critical determinants of several treatment successes were geographical location, type of gastric acid inhibitor, and treatment duration. The effectiveness of this treatment among Iranians was 73% lower compared to other geographical locations. Based on the results of this study, TE is improved by prolonged treatment and the use of omeprazole. The effectiveness of the 7-day OBNA regimen in the United States, Europe, and China was high, and the weighted mean of TE under these conditions was 95% (a confidence interval of 90-99%). The effectiveness of using the OBNA regimen was less than 80% with a weighted mean for a duration shorter than one week for its utilization in Iran (48). Accordingly, although the cost effectiveness of furazolidone (group A) was higher than that of clarithromycin (group B), the administration of furazolidone should be considered cautiously along with an investigation of all relevant variables and health and population conditions due to the higher effectiveness of clarithromycin (group B) versus furazolidone (group A).

One of the most important strengths of the present study is the use of the PERSIAN cohort study data (available at https://persiancohort.com/) as a strong, valid, and approved protocol among Middle Eastern countries. The large sample size, calculation of direct and indirect costs, and ICER as well as the study being conducted in a region with a high prevalence of gastrointestinal cancer are other strengths of this study. Failure to investigate the side effects of the drugs studied, lack of access to more participant economic data because of some related sensitivity, and recall bias about some other economic data are some of the limitations of the present study.

The present study showed that the cost effectiveness of *H. pylori *treatment with furazolidone was higher than that of clarithromycin and per increased effectiveness of 1%, less cost is paid compared to clarithromycin; however, the effectiveness of clarithromycin was significantly higher than that of furazolidone, affecting the decision on replacing this method with furazolidone. Therefore, due to the high prevalence of *H. pylori *and the economic conditions of the health system in Iran, such as facing drug sanctions and high direct treatment costs, the combination of furazolidone with amoxicillin 500 + omeprazole 20 + bismuth 120 can be a cost-effective choice between the two conventional treatment methods. Based on the results of this study, it can be recommended that furazolidone could replace clarithromycin to avoid the economic burden of *H. pylori *treatment. Accordingly, for policy-making and decision-making for the better choice, the future studies are recommended not only to consider the cost effectiveness of *H. pylori *treatment, but also to consider the side effects of taken drugs along with expenses after treatment.
